# Supplementary material for: Inhibition of RNA splicing is a novel therapeutic strategy for disruption of nuclear replicating viruses
Source: bioRxiv. 2026 Jun 27:2026.06.26.734849. Preprint. [Version 1] doi: 10.64898/2026.06.26.734849 (PMC13320946; doi:10.64898/2026.06.26.734849)
Supplement: 1 [file NIHPP2026.06.26.734849v1-supplement-1.pdf]

bioRxiv preprint doi: https://doi.org/10.64898/2026.06.26.734849; this version posted June 27, 2026. The copyright holder for this preprint (which was not certified by peer review) is the author/funder, who has granted bioRxiv a license to display the preprint in perpetuity. It is made available under a CC-BY 4.0 International license.

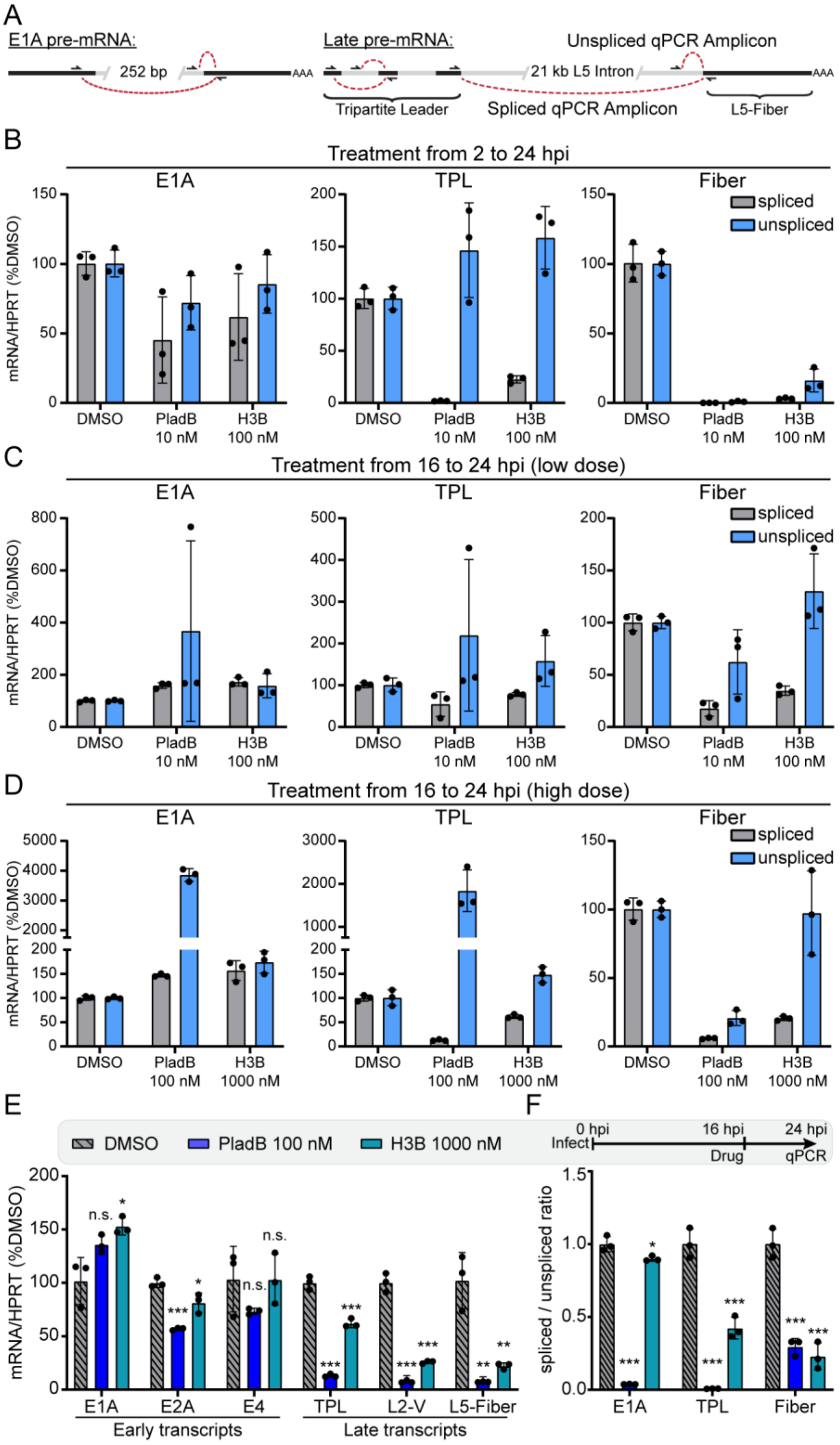

Supplementary Figure 1. Spliced-transcript aware PCR reveals specific effect of both low- and high-dose splicing inhibition. (A) Model showing representative transcripts and primer design to identify spliced and unspliced adenoviral early and late transcripts by qRT-PCR. **(B)** Individual spliced and unspliced E1A, TPL, and Fiber transcript results used to calculate the splicing ratio in Figure 2B. **(C)** Individual spliced and unspliced E1A, TPL, and Fiber transcript results used to calculate the splicing ratio in Figure 2D. **(D)** A549 cells were infected with Ad5 and treated with higher concentrations of PladB (100 nM, blue) or H3B (1000 nM, teal) at 16 hpi. Total RNA was harvested at 24 hpi and individual spliced and unspliced E1A, TPL, and Fiber transcripts are shown. **(E)** Total RNA from D was analyzed by qRT-PCR

bioRxiv preprint doi: https://doi.org/10.64898/2026.06.26.734849; this version posted June 27, 2026. The copyright holder for this preprint (which was not certified by peer review) is the author/funder, who has granted bioRxiv a license to display the preprint in perpetuity. It is made available under a CC-BY 4.0 International license.

for listed viral early and late transcripts and normalized to both cellular HPRT1 transcripts and DMSO-treated vehicle controls. Data points are shown for three biological replicates, bars depict mean, and error bars show standard deviation. **(F)** Data from D rearranged to show the splicing ratio of E1A, TPL, and Fiber. Significance was analyzed by unpaired two-tailed *t*-test, and displayed as P-value >0.5 (not-significant, n.s.), * P< 0.05, ** P< 0.01 and *** P< 0.001.

bioRxiv preprint doi: https://doi.org/10.64898/2026.06.26.734849; this version posted June 27, 2026. The copyright holder for this preprint (which was not certified by peer review) is the author/funder, who has granted bioRxiv a license to display the preprint in perpetuity. It is made available under a CC-BY 4.0 International license.

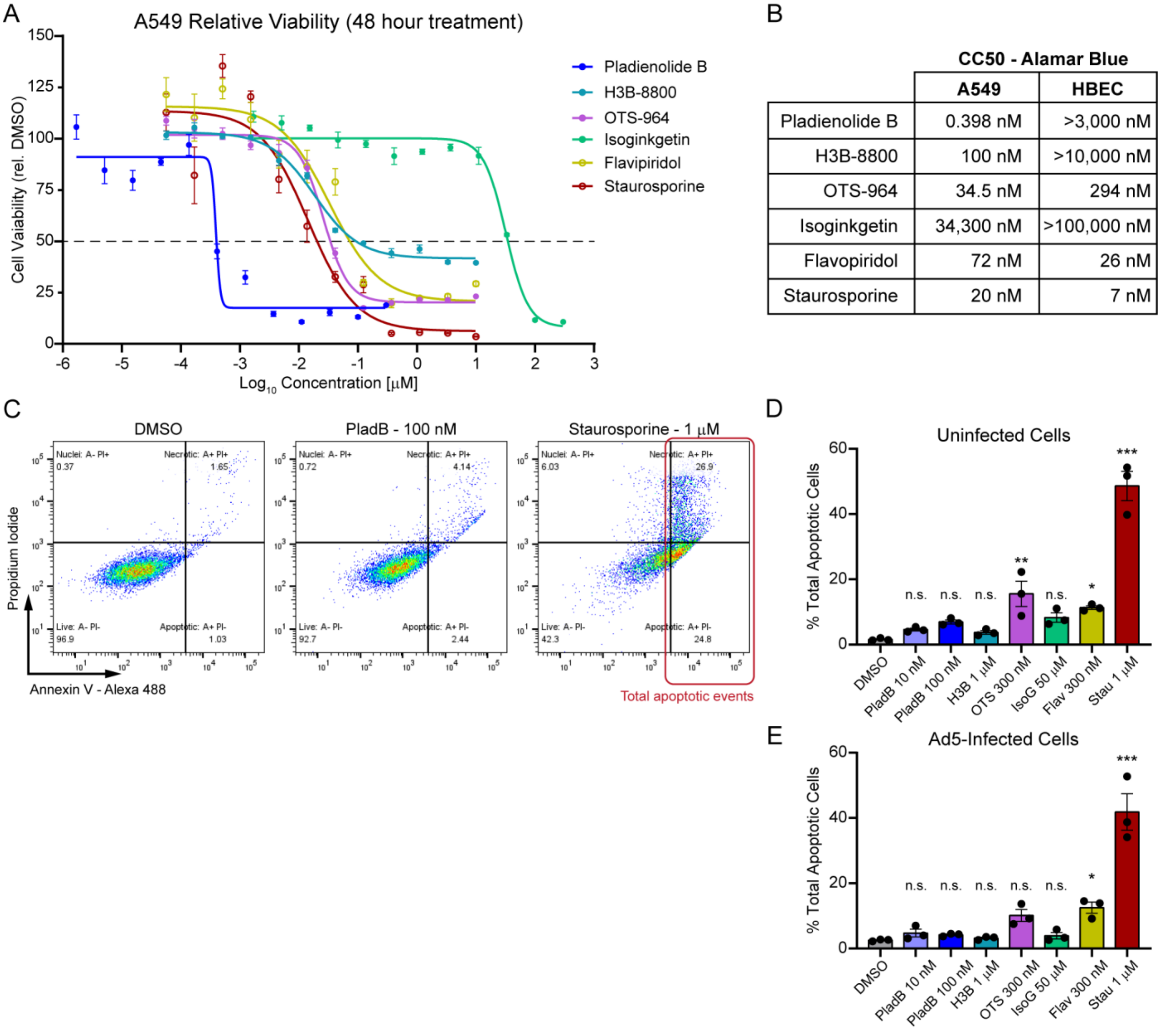

CC50 - Alamar Blue	A549	HBEC
Pladienolide B	0.398 nM	>3,000 nM
H3B-8800	100 nM	>10,000 nM
OTS-964	34.5 nM	294 nM
Isoginkgetin	34,300 nM	>100,000 nM
Flavopiridol	72 nM	26 nM
Staurosporine	20 nM	7 nM

Supplementary Figure 2. Low-dose splicing inhibitors do not cause overt cytotoxicity. (A) A549 cells were seeded in 96 well plates and treated with serial dilutions of PladB (blue), H3B (teal), OTS (purple), IsoG (green), flavopiridol (yellow), or staurosporine (red) and incubated for 48 hours. Relative cell viability was measured by Alamar blue fluorescence assay and normalized to DMSO vehicle control at 100% and media only at 0%. Points represent four independent biological replicates, and error bars denote standard deviation. Curve was fitted using a four parameter log(inhibitor) vs response formula. **(B)** Cytotoxic Concentration at 50% (CC50) values constructed using Alamar blue viability data from the 50% intercept of the calculated nonlinear fit curve for each drug for both A549 cells and the confluent HBECs assayed in Figure 5A. **(C)** Representative flow cytometry plots for Annexin V and propidium iodide-based cell apoptosis assay performed on uninfected A549 cells treated with the listed concentration of drugs for 24 hours. **(D)** Total apoptotic events (Annexin V^+, PI $^{+/-}$), as a percentage of all events, for uninfected A549 cells treated with the listed concentration of and type of drug for 24 hours. **(E)** Same as in D, but A549 cells were infected with Ad5 for two hours before subsequent 24 hour treatment with listed drugs and apoptosis assay performed a total of 26 hpi. Statistical significance for flow cytometry data was analyzed by lognormal ordinary one-way ANOVA, and displayed as P-value >0.5 (not-significant, n.s.), * P< 0.05, ** P< 0.01 and *** P< 0.001.

bioRxiv preprint doi: https://doi.org/10.64898/2026.06.26.734849; this version posted June 27, 2026. The copyright holder for this preprint (which was not certified by peer review) is the author/funder, who has granted bioRxiv a license to display the preprint in perpetuity. It is made available under a CC-BY 4.0 International license.

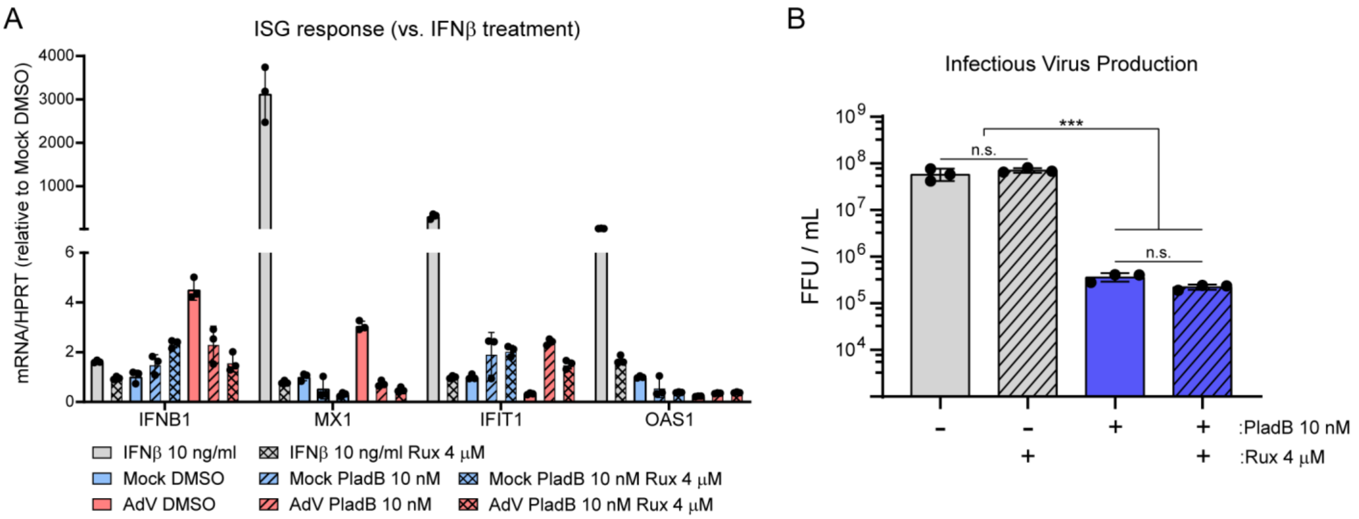

Supplementary Figure 3. Viral specific effects of splicing inhibition are independent from an interferon stimulated response. (A) A549 cells were mock-infected or Ad5-infected for two hours before treatment with either 10 nM PladB alone or 10 nM PladB plus 4 μM ruxolitinib (Rux). As a control, separate wells of uninfected A549 cells were treated with 10 ng/mL recombinant Interferon Beta (IFNβ) alone or in combination with concurrent 4 μM rux treatment. Twenty-four hours post infection total RNA was harvested and subjected to qRT-PCR for interferon beta transcripts or select interferon stimulated genes. Individual transcripts were normalized to internal housekeeping control HPRT and displayed as fold over the average mock DMSO-treated cells. Bars represent the average of three biological replicates, and error bars denote standard deviation. **(B)** A549 cells were infected with fluorescent Ad5 (E3mNG) and treated with 10 nM PladB alone, 4 μM Rux alone, or 10 nM PladB plus 4 μM Rux at 2 hpi. Total cell plus supernatant was collected at 48 hpi and released viruses used for virus progeny calculation via fluorescent forming unit (FFU). Data are presented as log-scale, points show the average of three biological replicates, and error bars show standard deviation. Significance was analyzed by unpaired two-tailed *t*-test (or lognormal ordinary one-way ANOVA for infectious titer data) and displayed as P-value >0.5 (not-significant, n.s.), * P< 0.05, ** P< 0.01 and *** P< 0.001.

bioRxiv preprint doi: https://doi.org/10.64898/2026.06.26.734849; this version posted June 27, 2026. The copyright holder for this preprint (which was not certified by peer review) is the author/funder, who has granted bioRxiv a license to display the preprint in perpetuity. It is made available under a CC-BY 4.0 International license.

Supplementary Table 1. List of primers used in this study

**AdV specific primers**	**Forward Primers (5' to 3')**	**Reverse Primers (5' to 3')**
E1A spliced	GTACCGGAGGTGATCGATCTTA	TCAGGCTCAGGTTCAGACA
E1A pre-mRNA	GGTGGGTTTGGTGTGGTAAT	
E4 common	GACAGGAAACCGTGTGGAATA	CACAGAGTACACAGTCCTTTCTC
E2A-DBP	GCCATTGCGCCCAAGAAGAA	CTGTCCACGATTACCTCTGGTGAT
TPL spliced	TTCCGCATCGCTGTCTG	CCGATCCAAGAGTACTGGAAAG
TPL pre-mRNA	GTCCAGGGTTTCCTTGATGAT	
L2-V	GAAAGGCGTCTAACCAGTCA	TGTAATCCTGCTCTTCCTTCTTC
L5-Fiber	GAAAGGCGTCTAACCAGTCA	AAAGGCACAGTTGGAGGAC
L5-Fiber per-mRNA	CATCCGCACCCACTATCTTC	
**Cell specific primers**	**Forward Primers (5' to 3')**	**Reverse Primers (5' to 3')**
HPRT	TGACACTGGCAAAACAATGCA	GGTCCTTTTCACCAGCAAGCT
IFNB1	CAGCATCTGCTGGTTGAAGA	CATTACCTGAAGGCCAAGGA
MX1	GGCCAGCAAGCGCATCT	TGGAGCATGAAGAACTGGATGA
IFIT1	GGATTCTGTACAATACACTAGAAACCA	CTTTTGGTTACTTTTCCCCTATCC
OAS1	GAAGGCAGCTCACGAAACC	AGGCCTCAGCCTCTTGTG
**Genomic primers**	**Forward Primers (5' to 3')**	**Reverse Primers (5' to 3')**
gTubulin	CCAGATGCCAAGTGACAAGAC	GAGTGAGTGACAAGAGAAGCC
DBP	GCCATTGCGCCCAAGAAGAA	CTGTCCACGATTACCTCTGGTGAT
		**Continues to next page**

bioRxiv preprint doi: https://doi.org/10.64898/2026.06.26.734849; this version posted June 27, 2026. The copyright holder for this preprint (which was not certified by peer review) is the author/funder, who has granted bioRxiv a license to display the preprint in perpetuity. It is made available under a CC-BY 4.0 International license.

**HSV1 specific primers**	**Forward Primers (5' to 3')**	**Reverse Primers (5' to 3')**
ICP4	GAAGTTGTGGACTGGGAAGG	GTTGCCGTTTATTGCGTCTT
ICP0 mature	GCGAGTACCCGCCGGCCTGA	CTCGAACAGTTCCGTGTCC
ICP0 pre-mRNA	GATCCAAAGGACGGACCCAG	GATTTCCCGCGTCAATCAGC
UL15 mature	CCCGAGTGGACCACGTTAAA	CTCGTCGACAAAGAGCAGGT
UL15 pre-mRNA	CCCACCCACATACACACACA	CTCCTCAAGCGATCCCGAAT
**Influenza specific primers**	**Forward Primers (5' to 3')**	**Reverse Primers (5' to 3')**
IAV-M2	CGAGGTCGAAACGCCTATCAGAAAC	TCAAGTGCAAGATCCCAATG
IAV-M1	ATTGGGACTCATCCTAGCTCC	
IAV-NEP	TGTCAAGCTTCCAGGACATAC	CTTCTCCAAGCGAATCTCTG
IAV-NS1	ACCATTGCCTTCTCTTCCAG	
**RT-PCR primers**	**Forward Primers (5' to 3')**	**Reverse Primers (5' to 3')**
DNAJB1	GAACCAAAATCACTTTCCCCAAGGAAGG	AATGAGGTCCCCACGTTTCTCGGGTGT
BRD2	CAAAATTATAAAACAGCCTATGGACATG	TTTTCCAGCGTTTGTGCCATTAGGA
GAPDH	CAGAACATCATCCCTGCCTCTACT	GCCGAGCTTCCCGTTCA
